# Adherence to the Mediterranean diet and mortality in cancer survivors: a Nationwide study with mediation and subgroup analyses

**DOI:** 10.3389/fnut.2025.1607522

**Published:** 2025-09-16

**Authors:** Bowen Zha, Lizhou Dou, Chen Zhang, Shun He, Guiqi Wang

**Affiliations:** Department of Endoscopy, National Cancer Center/National Clinical Research Center for Cancer/Cancer Hospital, Chinese Academy of Medical Sciences and Peking Union Medical College, Beijing, China

**Keywords:** Mediterranean diet, cancer survivors, all-cause mortality, NHANES, cox regression, mediation analysis

## Abstract

**Background:**

Mediterranean diet (MD) adherence is linked to improved health outcomes, yet evidence among cancer survivors remains limited. This study investigated the association between Mediterranean Diet Score (MDS) adherence and all-cause mortality among cancer survivors.

**Methods:**

We analyzed data from 2,669 cancer survivors participating in the National Health and Nutrition Examination Survey (2005–2018). Dietary adherence was assessed using the MDS based on 2-day dietary recalls. Multivariable Cox regression, mediation, subgroup, and sensitivity analyses were conducted.

**Results:**

Higher MDS adherence was significantly associated with reduced all-cause mortality (hazard ratio (HR) = 0.845, 95% confidence interval (CI): 0.779–0.917, *p* < 0.001), with a linear dose–response trend. Mediation analysis showed that red blood cell distribution width and neutrophils explained 18.5 and 7.8% of the association, respectively. Subgroup analyses revealed stronger protective effects in females, older adults, individuals with lower BMI or higher socioeconomic status, smokers, drinkers, and survivors of digestive, urinary, and skin cancers. Sensitivity analyses confirmed the robustness of the findings.

**Conclusion:**

Greater adherence to the Mediterranean diet is associated with lower all-cause mortality in cancer survivors, partly mediated by inflammatory biomarkers. Integrating Mediterranean dietary counseling into survivorship care may help improve long-term outcomes.

## Introduction

Cancer survivorship has become a critical focus in public health worldwide (1–3). Advances in diagnosis and treatment have improved survival rates, yet long-term mortality and quality of life remain significant concerns. Among modifiable post-diagnosis factors, dietary patterns have garnered increasing attention for their potential role in improving prognosis and reducing the risk of recurrence and comorbidities (4–8).

The Mediterranean diet (MD), characterized by high consumption of vegetables, fruits, legumes, whole grains, fish, nuts, and olive oil, along with a low intake of red meat and saturated fats, has been extensively studied for its cardiometabolic and anti-inflammatory benefits (9–14). The Mediterranean Diet Score (MDS), a composite score reflecting adherence to the MD, has been linked to a lower risk of chronic diseases and all-cause mortality in general populations (15, 16). However, its prognostic implications for cancer survivors remain less well established.

However, most existing studies have focused primarily on breast, colorectal, or prostate cancer survivors, leaving other cancer types underrepresented in the literature (17–22). Survivors of cancers such as lung, digestive system, reproductive system, and hematologic malignancies, which often carry worse prognoses and a greater systemic burden, have been largely excluded or insufficiently powered in prior cohort studies. This limits the generalizability of findings and impedes the development of broad dietary recommendations for the cancer survivor population as a whole. Some prospective studies have reported improved survival and reduced recurrence with higher MDS adherence, while others have found null associations (23). These discrepancies may arise from differences in cancer types, dietary assessment methods, or follow-up durations. Furthermore, most studies have not addressed potential biological mechanisms linking MDS to outcomes. Inflammation and oxidative stress are known to play central roles in cancer progression and survivorship health, yet few studies have explored these pathways as potential mediators (24, 25).

Therefore, we aimed to investigate the association between MDS adherence and all-cause mortality in a nationally representative cohort of U.S. cancer survivors from the NHANES 2005–2018 cycles. Leveraging a robust analytical framework, we used Cox proportional hazards models, restricted cubic splines, and stratified subgroup analyses to assess the dose–response and effect modification. Additionally, we conducted mediation analyses to explore potential inflammatory pathways, focusing on biomarkers such as red cell distribution width (RDW) and neutrophil count (NEU), which may partially explain the observed associations.

## Methods

### Participant selection

**Figure 1:**
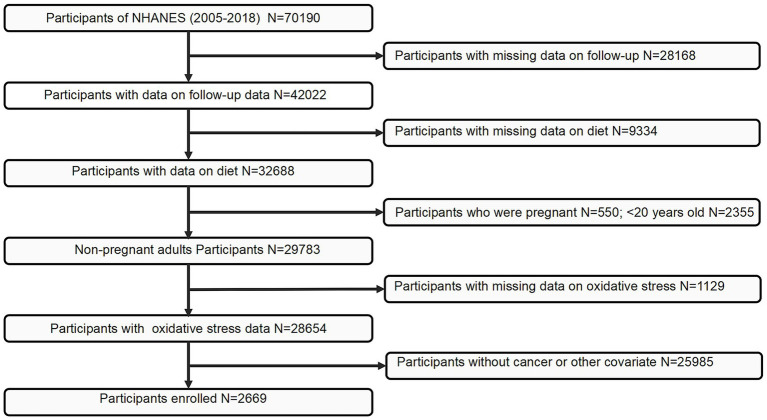
Flowchart of the study population. NHANES, National Health and Nutrition Examination Survey.

The present study utilized data from the National Health and Nutrition Examination Survey (NHANES), administered by the National Center for Health Statistics (NCHS). NHANES cycles prior to 2005 were excluded due to inconsistencies in dietary data collection protocols and the limited availability of key variables required for constructing the MDS and covariate adjustments. Initially, 70,190 individuals from the 2005–2018 cycles were considered. Participants completed a standardized questionnaire that included the item: “Has a medical practitioner or healthcare provider ever informed you that you had cancer or a malignancy of any kind?” Individuals who answered affirmatively were categorized as cancer survivors. Following a rigorous screening process, 2,669 participants were included in the final analysis. Details of the participant selection process are illustrated in Figure 1.

### Assessment of the Mediterranean diet

Following interviews conducted by specially trained personnel, dietary data were collected using computer-assisted 24-h dietary recall interviews. Dedicated team members were responsible for data review and processing. The United States Department of Agriculture has repeatedly simulated and refined this process to ensure stringent quality control. In this study, dietary data from the first and second days were utilized. While 24-h recalls are subject to recall bias, NHANES uses standardized protocols, trained interviewers, and repeated measures to improve data reliability. This method has been validated for assessing dietary patterns in large populations. Although cancer stage and treatment data were unavailable, our analyses were adjusted for multiple health-related and sociodemographic variables, aligning with the study’s population-level objectives.

The Mediterranean Diet Score (MDS) is an index designed to assess adherence to the Mediterranean diet (26). We used a modified MDS adapted for the dataset in the study (27). The score comprises 10 components: one point is assigned for intakes equal to or above the median for non-refined cereals, legumes, fruits and nuts, vegetables, and fish and for a favorable ratio of monounsaturated to saturated fatty acids. The median cutoffs for each MDS component were derived from the study sample of cancer survivors. Since MDS is a relative index, absolute intake values are not used for scoring. To facilitate interpretability and examine dose–response patterns, participants were categorized into quartiles (Q1–Q4) based on their MDS distribution within the analytic sample. Although group sizes were not perfectly equal due to score discreteness, quartile-based classification is a standard method in nutritional epidemiology.

### Ascertainment of mortality

All-cause mortality rate was determined through probabilistic matching of unique identifiers with the National Death Index. Participants lacking a corresponding record were assumed to be alive as of the specified cutoff date. Follow-up duration was measured in person-years from the baseline assessment until death or the conclusion of the follow-up period on 31 December 2019. In accordance with the guidelines of the International Classification of Diseases, 10th Revision (ICD-10), deaths due to all causes were identified. All-cause mortality encompassed deaths attributable to cardiovascular diseases, malignant neoplasms, and other causes.

### Covariates

This study incorporated several covariate factors, as identified by previous research, for comprehensive analysis and discussion. The covariates included age, sex, ethnicity, education, poverty-to-income ratio (PIR), body mass index (BMI), smoking status, alcohol consumption, diabetes, and hypertension. Ethnicity was categorized as Mexican American, other Hispanic, non-Hispanic white, non-Hispanic Black, and other ethnicities. Education levels were stratified into less than 9th grade, 9th–11th grade, high school, college, and graduate-level or higher. Smoking status was defined as having smoked more than 100 cigarettes over a lifetime, while alcohol consumption was determined by the annual intake equivalent to 12 ounces of beer, 5 ounces of wine, or 1.5 ounces of liquor. Diabetes was diagnosed based on medical history and fasting blood glucose levels, and hypertension was ascertained through a combination of medical history and multiple blood pressure measurements. NHANES does not collect data on cancer site, diagnosis date, stage, or treatment modalities in a systematic manner. As such, these cancer-specific covariates could not be included and are acknowledged as a limitation.

### Statistical analyses

Participants were divided into four groups based on their MDS, and baseline characteristics were summarized using frequencies (percentages) for categorical variables and means (standard errors) for continuous variables.

To evaluate the association between MDS and all-cause mortality in cancer survivors, multivariable Cox regression models were used. Hazard ratios (HRs) along with their corresponding 95% confidence intervals (CIs) were calculated to quantify these associations. Three progressively adjusted models were constructed: Model 1 was adjusted solely for age and sex; Model 2 further incorporated ethnicity, education, PIR, and BMI; and Model 3 additionally controlled for mortality risk factors, including diabetes, hypertension, alcohol consumption, and smoking status. Furthermore, a restricted cubic spline (RCS) analysis was performed to explore the relationship between MDS and prognosis. Consistent with common epidemiologic practice, the node of RCS was set to four (28, 29).

Previous studies have demonstrated that adherence to the Mediterranean diet is closely associated with markers of inflammation and oxidative stress, which are critical contributors to the pathogenesis of cancer prognosis (30–33). In line with these findings, a mediation analysis was conducted to investigate the potential mediating roles of neutrophils (NEU), lymphocytes (LYM), red blood cell distribution width (RDW), alkaline phosphatase (ALP), *γ*-glutamyltransferase (GGT), total bilirubin (TB), and uric acid (UA) in the relationship between MDS and prognosis. This analysis aimed to elucidate the underlying biological mechanisms and identify potential targets for intervention by quantifying the average causal mediation effects (ACMEs), average direct effects (ADEs), total effect, and the proportion of the mediating effect relative to the total effect.

To further explore the relationships, subgroup analyses were performed by stratifying participants according to age (20–65 years vs. ≥65 years), sex (male vs. female), smoking status (smoking vs. non-smoking), alcohol consumption (drinking vs. non-drinking), BMI (<median vs. ≥median), poverty income ratio (PIR; <median vs. ≥median), and type of malignancy (urinary system, digestive system, skin and soft tissues, lungs and mediastinal organs, breast, and reproductive system, or others). Multivariable Cox regression analyses were conducted within each subgroup. Interaction terms were tested for each subgroup to evaluate effect modification.

To assess the robustness of our findings, we performed three sensitivity analyses: (1) excluding participants who died within 1 year of the interview, (2) sequentially removing data from each NHANES cycle (each spanning 2 years) to evaluate the stability of the results, and (3) utilizing the first-day dietary data to validate the association, ensuring that the observed relationship was not affected by the averaging method of the 2-day data. Analyses were conducted using R (Revision 4.4.1), with statistical significance defined as a *p*-value of < 0.05.

## Result

### Baseline characteristics

The baseline characteristics of the study population across MDS quartiles are presented in Table 1. The mean age of the participants was 66.0 years (SD: 13.8), with slight variations across quartiles. Females accounted for 52.6% of the overall cohort, with a higher proportion observed in Q2 and Q3. The majority of participants were non-Hispanic white (69.8%), although Q4 showed a modest increase in the representation of Hispanic individuals. Educational attainment varied substantially across groups, with the highest proportion of participants holding graduate-level education in Q4 (34.9%) and the lowest in Q1 (17.2%).

**Table 1:** Baseline characteristics of participants stratified by MDS quartile.

Variables	Q1 (*N* = 529)	Q2 (*N* = 477)	Q3 (*N* = 548)	Q4 (*N* = 1,115)	Overall (*N* = 2,669)
Age (years)	65.6 (14.1)	67.2 (13.7)	66.6 (13.7)	65.5 (13.6)	66.0 (13.8)
Sex					
Male, %	274(51.8%)	220 (46.1%)	235 (42.9%)	535 (48.0%)	1,264 (47.4%)
Female, %	255 (48.2%)	257 (53.9%)	313 (57.1%)	580 (52.0%)	1,405 (52.6%)
Race/ethnicity, %					
Mexican American	25 (4.7%)	27 (5.7%)	38 (6.9%)	81 (7.3%)	171 (6.4%)
Other Hispanic	26 (4.9%)	22 (4.6%)	31 (5.7%)	78 (7.0%)	157 (5.9%)
Non-Hispanic white	366 (69.2%)	338 (70.9%)	395 (72.1%)	764 (68.5%)	1863 (69.8%)
Non-Hispanic black	94 (17.8%)	78 (16.4%)	60 (10.9%)	119 (10.7%)	351 (13.2%)
Other races	18 (3.4%)	12 (2.4%)	24 (4.4%)	73 (6.5%)	127 (4.7%)
Education level, %					
<9th grade	49 (9.3%)	36 (7.5%)	64 (11.7%)	86 (7.7%)	235 (8.8%)
9–11th grade	67 (12.7%)	73 (15.3%)	66 (12.0%)	105 (9.4%)	311 (11.7%)
High school	155 (29.3%)	117 (24.5%)	121 (22.1%)	197 (17.7%)	590 (22.1%)
College	167 (31.6%)	141 (29.6%)	170 (31.0%)	338 (30.3%)	816 (30.6%)
Graduate or above	91 (17.2%)	110 (23.1%)	127 (23.2%)	389 (34.9%)	717 (26.8%)
Poverty impact ratio	2.5 (1.5)	2.6 (1.6)	2.7 (1.6)	3.1(1.6)	2.8 (1.6)
Body mass index (kg/m^2^)	30.1 (7.4)	29.3 (6.2)	29.6 (6.6)	28.4 (6.1)	29.1 (6.5)
Hypertension					
With hypertension	319 (60.3%)	274 (57.4%)	325 (59.3%)	616 (55.2%)	1,534 (57.5%)
Without hypertension	210 (39.7%)	203 (42.6%)	223 (40.7%)	499 (44.8%)	1,135 (42.5%)
Diabetes					
With diabetes	95 (18.0%)	91 (19.0%)	134 (24.5%)	206 (18.5%)	526 (19.7%)
Without diabetes	434 (82.0%)	386 (81.0%)	414 (75.5%)	909 (81.5%)	2,143 (79.2%)
Smoking status					
Smoking	323 (61.1%)	254 (53.2%)	291 (53.1%)	574 (51.5%)	1,442 (54.0%)
No-smoking	206 (38.9%)	223 (46.8%)	257 (46.9%)	541 (48.5%)	1,227 (46.0%)
Alcohol consumption					
Drinking	301 (56.9%)	270 (56.6%)	312 (56.9%)	673 (60.4%)	1,556 (58.3%)
No-drinking	228 (43.1%)	207 (43.4%)	236 (43.1%)	442 (39.6%)	1,113 (41.7%)
Overall survival (months)	73.5 (44.8)	77.2 (46.3)	81.3 (47.5)	81.6 (45.8)	79.1 (46.2)

Q1–Q4 represent increasing quartiles of MDS, from lowest to highest.

A decreasing trend in BMI was observed across MDS quartiles, from 30.1 kg/m^2^ in Q1 to 28.4 kg/m^2^ in Q4. Similarly, the prevalence of hypertension decreased with increasing MDS adherence, from 60.3% in Q1 to 55.2% in Q4. Smoking prevalence was highest in Q1 (61.1%) and gradually decreased across higher quartiles. Alcohol consumption was relatively consistent across all groups, although it was slightly higher in Q4 (60.4%). Mean overall survival increased progressively with higher MDS, from 73.5 months in Q1 to 81.6 months in Q4. Details are shown in Table 1.

### Survival analysis

**Figure 2:**
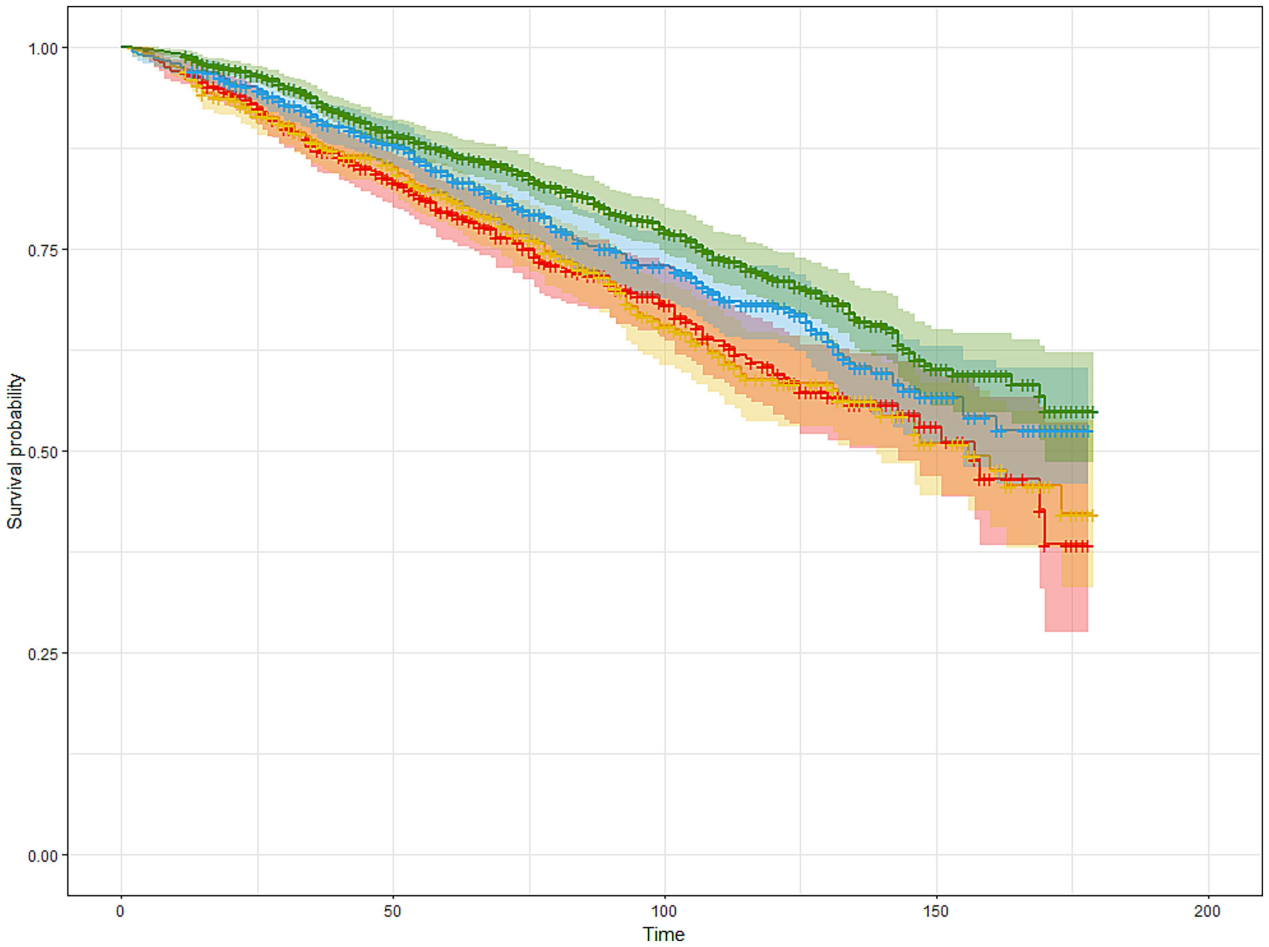
The Kaplan–Meier estimated survival curves of participants with cancer based on MDS quartiles. Participants were stratified into four groups (Q1–Q4) based on sex-specific quartiles of MDS. The horizontal axis represents follow-up time (months, 0–200), and the vertical axis shows survival probability. Curves were adjusted for age, sex, ethnicity, education level, PIR, BMI, smoking status, alcohol consumption, hypertension, and diabetes. Red, blue, yellow, and green lines correspond to Q1, Q2, Q3, and Q4 of MDS, respectively.

As shown in Table 2 and Figure 2, higher MDS was significantly associated with improved survival in cancer survivors. In the minimally adjusted model (Model 1), individuals in Q4 had a 29.6% lower risk of mortality compared to those in Q1 (HR = 0.704, 95% CI: 0.574–0.864, *p* < 0.001). This inverse association remained robust after further adjustment for socioeconomic factors in Model 2 (HR = 0.690, 95% CI: 0.557–0.855, *p* < 0.001), and lifestyle-related risk factors in Model 3 (HR = 0.672, 95% CI: 0.542–0.833, *p* < 0.001).

**Table 2:** Association between MDS and prognosis in cancer survivors.

MDS/Quartiles	Model 1		Model 2		Model 3	
	HR(SE)	*p* value	HR(SE)	*p* value	HR(SE)	*p* value
MDS	0.844 (0.781, 0.912)	<0.001	0.853 (0.787, 0.924)	<0.001	0.845 (0.779, 0.917)	<0.001
Q1	Ref	–	Ref	–	Ref	–
Q2	0.945 (0.753, 1.185)	0.621	0.932 (0.735, 1.182)	0.561	0.923 (0.727, 1.171)	0.508
Q3	0.767 (0.613, 0.961)	0.021	0.763 (0.604, 0.964)	0.023	0.737 (0.583, 0.931)	0.011
Q4	0.704 (0.574, 0.864)	<0.001	0.690 (0.557, 0.855)	<0.001	0.672 (0.542, 0.833)	<0.001

Model 1: Adjusted for age, sex, race, and education level; Model 2: additionally adjusted for PIR and BMI; Model 3: further adjusted for smoking, alcohol consumption, hypertension and diabetes. The first column presents hazard ratios per 1-point increase in the Mediterranean Diet Score as a continuous variable. Q1–Q4 represent increasing quartiles of MDS, from lowest to highest. HR, hazard ratio; CI, confidence interval; SE, standard error.

While Q2 and Q3 showed a trend toward lower mortality risk compared to Q1, only Q3 reached statistical significance in Model 3 (HR = 0.737, 95% CI: 0.583–0.931, *p* = 0.011), suggesting a dose–response relationship primarily driven by higher adherence to MDS.

**Figure 3:**
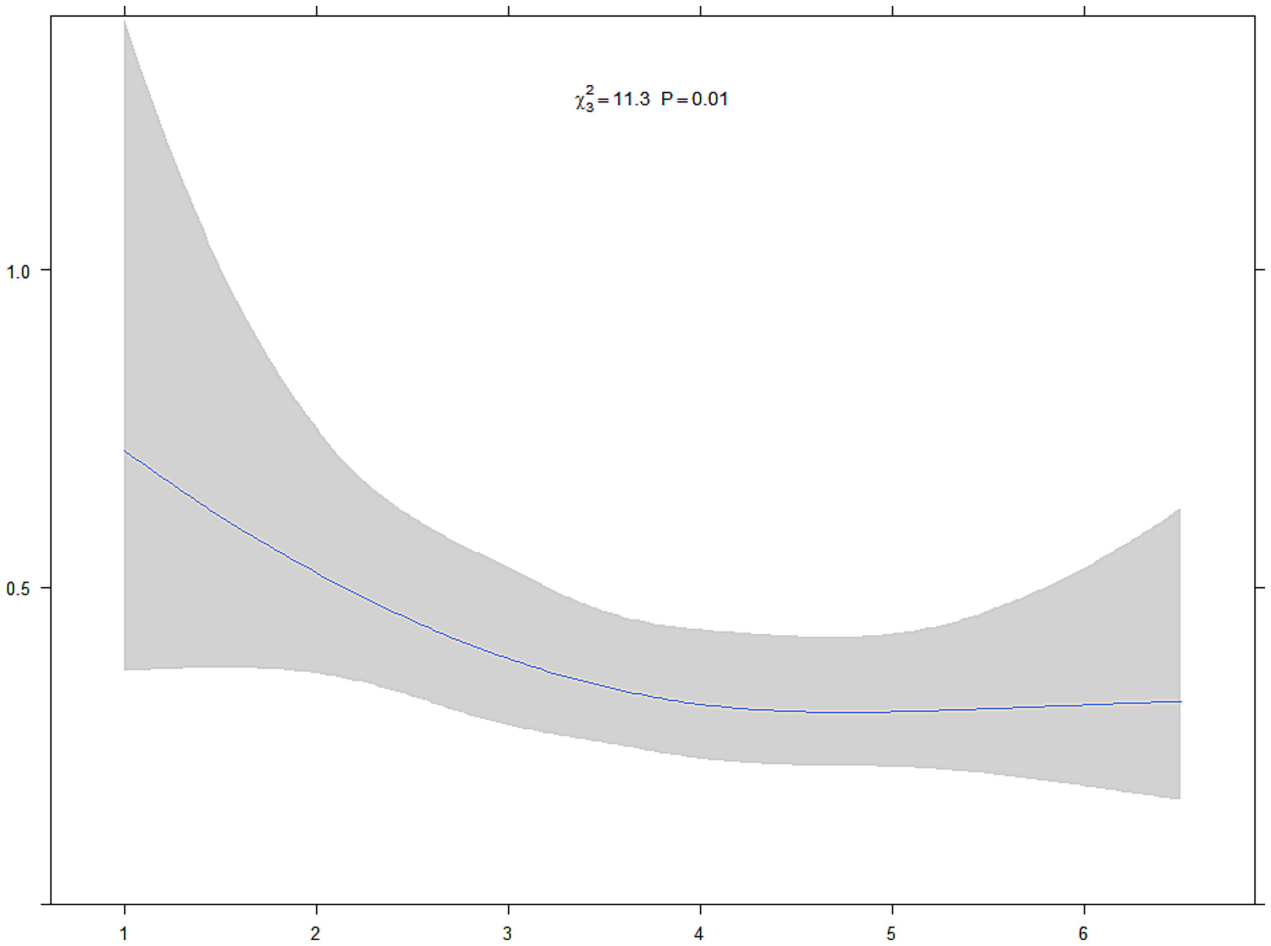
The restricted cubic spline estimated survival curves of participants with cancer based on MDS quartiles. The horizontal axis represents the MDS, and the vertical axis displays the estimated HR for all-cause mortality. The blue line indicates the adjusted HR values, while the shaded area represents the 95% CI. The reference value (HR = 1.0) corresponds to the median MDS. Estimates were derived from Cox proportional hazards models adjusted for age, sex, ethnicity, education level, PIR, BMI, smoking status, alcohol consumption, hypertension, and diabetes.

In addition, RCS analysis indicated a significant linear association between MDS and all-cause mortality (*p* for overall association < 0.001), without evidence of non-linearity (*p* for non-linearity = 0.29), as shown in Figure 3. These findings suggest that increasing adherence to the MDS is linearly associated with reduced mortality risk among cancer survivors.

### Mediation analysis

Mediation analysis was performed to explore the potential biological mechanisms linking MDS to prognosis, with inflammatory and oxidative stress-related biomarkers considered as mediators. As shown in Table 3, the total effect of MDS on prognosis was estimated at −0.024. Among the biomarkers assessed, RDW exhibited the strongest mediating effect, with an ACME of −0.004, accounting for 18.5% of the total effect. NEU also showed a modest mediation proportion of 7.82% (ACME = −0.002). TB and UA contributed to 9.01 and 4.13% of the total effect, respectively. Conversely, LYM, ALP, and GGT showed minimal mediation, with ACME values near zero and proportion mediated below 3.5%. These results suggest that certain inflammatory markers, particularly RDW and NEU, may partially mediate the protective association between MDS and mortality risk among cancer survivors.

**Table 3:** Mediation effects of inflammatory markers on the association between mds and prognosis in cancer survivors.

Mediate	ACME	ADE	Total effect	Prop mediated (%)
NEU	−0.002	−0.022	−0.024	7.821
LYM	0.000	−0.024	−0.024	0.023
RDW	−0.004	−0.020	−0.024	18.51
ALP	0.000	−0.023	−0.024	3.420
GGT	0.000	−0.024	−0.024	0.073
UA	−0.001	−0.023	−0.024	4.132
TB	−0.002	−0.022	−0.024	9.011

Models were adjusted for age, sex, race, education level, poverty-to-income ratio, BMI, smoking status, alcohol consumption, hypertension, and diabetes. ACME, average causal mediation effect; ADE, average direct effect; ALP, alkaline phosphatase; GGT, gamma-glutamyltransferase; LYM, lymphocyte count; NEU, neutrophil count; RDW, red blood cell distribution width; TB, total bilirubin; UA, uric acid.

### Subgroup analysis

Subgroup analyses demonstrated that the inverse association between MDS and all-cause mortality was largely consistent across the demographic and clinical strata, with several notable differences in effect magnitude. Both male and female participants exhibited significant protective associations, with a stronger effect observed in female participants (HR = 0.606, 95% CI: 0.418–0.880) than in male participants (HR = 0.713, 95% CI: 0.567–0.931). The association was more pronounced among participants aged ≥65 years (HR = 0.696, 95% CI: 0.546–0.886) compared to their younger counterparts, where the trend did not reach statistical significance.

Greater benefits were also observed in current smokers and drinkers, suggesting that adherence to the Mediterranean diet may mitigate some of the adverse effects associated with these behaviors. Individuals with lower BMI and higher socioeconomic status derived more pronounced survival benefits.

Stratification by cancer type revealed heterogeneity in effect size. The strongest inverse associations were observed among survivors of digestive system, urinary system, and skin/soft tissue cancers, whereas associations were weaker or non-significant in other cancer types. These patterns are summarized in Table 4 and Supplement Table 1.

**Table 4:** Subgroup analysis of effect of MDS with the prognosis of participant with cancer.

Subgroup	MDS [HR (95% CI)]	Q1	Q2 [HR (95% CI)]	Q3 [HR (95% CI)]	Q4 [HR (95% CI)]
Gender					
Male	0.840 (0.761, 0.927)	Ref	0.939 (0.900, 1.279)	0.706 (0.521, 0.957)	0.713 (0.567, 0.931)
Female	0.858 (0.745, 0.966)	Ref	0.840 (0.568, 1.241)	0.746 (0.501, 1.098)	0.606 (0.418, 0.880)
Age					
20–65	0.881 (0.718, 1.080)	Ref	0.846 (0.472, 1.514)	0.760 (0.412, 1.401)	0.838 (0.502, 1.401)
≥65	0.860 (0.786, 0.941)	Ref	0.968 (0.741, 1.262)	0.780 (0.601, 0.983)	0.696 (0.546, 0.886)
Type of malignancy					
Digestive system	0.732 (0.583, 0.918)	Ref	1.183 (0.618, 2.267)	0.649 (0.335, 1.258)	0.478 (0.250, 0.910)
Urinary system	0.803 (0.683, 0.946)	Ref	0.988 (0.613, 1.593)	0.620 (0.390, 0.986)	0.682 (0.453, 0.901)
Reproductive system	0.947 (0.639, 1.404)	Ref	1.332 (0.518, 3.429)	0.849 (0.290, 2.486)	0.998 (0.385, 1.185)
Breast	0.974 (0.739, 1.284)	Ref	0.750 (0.371, 1.514)	0.629 (0.309, 1.281)	0.629 (0.322, 1.230)
Skin and soft tissues	0.890 (0.785, 0.974)	Ref	0.931 (0.600, 1.447)	0.869 (0.568, 1.326)	0.664 (0.445, 0.990)
Others	0.947 (0.743, 1.207)	Ref	0.829 (0.398, 1.730)	0.916 (0.442, 2.048)	1.011 (0.545, 1.875)

Models were adjusted for age, sex, race, education level, poverty-to-income ratio, BMI, smoking status, alcohol consumption, hypertension, and diabetes. The first column presents hazard ratios per 1-point increase in the Mediterranean Diet Score as a continuous variable. Q1–Q4 represent increasing quartiles of MDS, from lowest to highest.

### Sensitivity analysis

To evaluate the robustness of the findings, multiple sensitivity analyses were conducted. First, participants who died within 1 year of the interview were excluded, and the analysis was repeated. Second, data from each NHANES cycle were sequentially removed to assess the stability of the effect estimates. Across all scenarios, the inverse association between MDS and all-cause mortality remained consistent. Additionally, the results obtained from different model specifications and methodological approaches were largely similar, further supporting the reliability of the main findings.

## Discussion

In this nationally representative cohort of cancer survivors from the NHANES dataset, we found that greater adherence to the MDS was significantly associated with reduced all-cause mortality. This association remained robust across progressively adjusted Cox regression models, was consistent in multiple subgroup and sensitivity analyses, and exhibited a linear dose–response pattern in RCS modeling. Furthermore, the mediation analysis suggested that specific inflammatory and oxidative stress-related biomarkers—particularly RDW and NEU—may partially mediate this relationship. While several prior NHANES-based studies have linked dietary patterns to mortality in the general population or patients with cardiometabolic diseases, few have focused specifically on cancer survivors. Moreover, most existing analyses have not explored the underlying biological mechanisms. By applying mediation analysis and incorporating inflammatory biomarkers such as RDW and NEU, our study offers mechanistic insights into how dietary adherence may affect survival in this high-risk population.

Our results align with previous findings that link MDS to improved survival in the general populations and among individuals with chronic diseases such as cardiovascular disease, diabetes, and metabolic syndrome (34–37). However, studies specifically focusing on cancer survivors remain limited. The current analysis highlights that, even among individuals who have undergone the physiologic burden of malignancy and its treatment, dietary quality may serve as an important prognostic indicator. This finding underscores the potential of dietary interventions as a modifiable post-diagnosis factor that may influence long-term outcomes in cancer survivorship.

The biological plausibility of these findings is supported by several mechanisms. The Mediterranean diet is rich in anti-inflammatory and antioxidant nutrients, such as monounsaturated fats, polyphenols, dietary fiber, and omega-3 fatty acids, all of which have been shown to modulate key pathways involved in carcinogenesis, immune regulation, and systemic inflammation (13, 38, 39). In the mediation analysis, RDW accounted for 18.5% of the total effect of MDS on mortality, followed by NEU (7.8%) and TB (9.0%). These biomarkers have previously been associated with poor prognosis in various cancers and may reflect systemic inflammation, oxidative stress, or bone marrow dysfunction (40–45). Although RDW, NEU, and TB were found to partially mediate the association between MDS and mortality, the overall mediation proportion was modest. This finding suggests that other unmeasured pathways may play a more prominent role in this relationship.

The consistency of the association across subgroups further strengthens the robustness of the results. Notably, the inverse relationship between MDS and mortality was more pronounced among older adults, females, individuals with lower BMI, and those with higher socioeconomic status, as reflected by PIR. These interactions may reflect differential susceptibility to the benefits of dietary quality, possibly due to underlying metabolic or immunologic differences. The stronger protective effect observed in smokers and drinkers is particularly noteworthy, suggesting that dietary modification may mitigate some of the adverse health risks associated with these behaviors (46, 47). These findings provide compelling evidence that, even among high-risk populations, dietary quality remains an actionable target. Biologically, this may be explained by the antioxidant and anti-inflammatory properties of the Mediterranean diet, which could counteract oxidative stress and systemic inflammation induced by smoking or alcohol consumption. Previous research has shown that high adherence to anti-inflammatory diets significantly lowers inflammatory biomarkers in high-risk populations, including smokers, lending support to this hypothesis. Nonetheless, residual confounding cannot be ruled out, as individuals with healthy dietary habits within these high-risk groups may also differ in other unmeasured lifestyle or health-related factors. Thus, these findings should be interpreted with caution and warrant further investigation in stratified or intervention-based studies.

Stratified analyses by cancer type revealed that the association between MDS and survival was strongest among patients with digestive system, urinary system, and skin and soft tissue malignancies. This may be due to the direct interaction of diet with gastrointestinal mucosal integrity, microbiota composition, and systemic inflammation, which are known to influence both local and systemic carcinogenic processes (48–50). The less consistent findings among reproductive, breast, and other cancer types may reflect smaller sample sizes or the influence of hormonal and genetic factors that attenuate the impact of dietary patterns. Additionally, the wide confidence intervals observed in several of these subgroups suggest limited statistical power, and the findings should therefore be interpreted as exploratory.

While the robustness of our findings was supported by sensitivity analyses and consistent subgroup patterns, the potential for reverse causality cannot be entirely excluded. It is possible that cancer survivors with better baseline health or greater health awareness were more likely to adhere to a Mediterranean-style diet, leading to an overestimation of its protective effect. To address this concern, we performed a sensitivity analysis excluding participants who died within 1 year of follow-up to reduce the likelihood of bias from pre-existing illness. Nonetheless, prospective or interventional studies are needed to establish temporality and causality with greater confidence.

Nonetheless, this study has several limitations. Residual confounding cannot be entirely ruled out despite adjustment for a wide range of covariates. Dietary data were based on 24-h recall interviews, which may not accurately capture long-term dietary patterns and are subject to recall and reporting bias. Moreover, cancer stage, treatment modalities, and time since diagnosis were not available in the NHANES dataset, limiting the ability to stratify analyses by disease severity or treatment status. Finally, although mediation analysis provides insights into potential biological pathways, it cannot fully disentangle complex interactions, and the proportion of mediation was relatively modest. Cancer-specific mortality outcomes, which could have provided more targeted insights, were not analyzed due to limited ICD-10 resolution and small event counts for specific cancer types.

In conclusion, higher adherence to the Mediterranean diet is independently associated with improved survival among cancer survivors in the United States. These findings highlight the potential of dietary quality as a modifiable prognostic factor in cancer survivorship and support the incorporation of Mediterranean dietary principles into post-diagnosis lifestyle recommendations. Specifically, dietary counseling interventions that emphasize Mediterranean components could be integrated into routine survivorship care, particularly targeting high-risk groups such as smokers, drinkers, or individuals with digestive or urinary system malignancies. Future prospective and interventional studies—including randomized controlled trials—are needed to confirm causality, evaluate dietary responsiveness across different cancer types, and determine the long-term efficacy of tailored nutritional interventions in improving survivorship outcomes.

## Data Availability

The raw data supporting the conclusions of this article will be made available by the authors, without undue reservation.
