# Supplementary material for: Adherence to the Mediterranean diet and mortality in cancer survivors: a Nationwide study with mediation and subgroup analyses
Source: Front Nutr. 2025 Sep 16;12:1607522. doi: 10.3389/fnut.2025.1607522 (PMC12479294; doi:10.3389/fnut.2025.1607522)
Supplement: Supplementary file 1 [file Table_1.DOCX]

Supplement 1 Subgroup analysis of effect of MDS with the prognosis of participant with Cancer

|  | MDS [HR (95% CI)] | Q1 | Q2 [HR (95% CI)] | Q3 [HR (95% CI)] | Q4 [HR (95% CI)] |
| --- | --- | --- | --- | --- | --- |
| Alcohol consumption |  |  |  |  |  |
| Drinking | 0.738(0.624,0.873) | Ref | 0.921(0.679,1.250) | 0.730(0.563,0.947) | \| 0.482(0.318,0.729) \| \| --- \| |
| No-drinking | 0.873(0.794,0.959) | Ref | 0.932(0.626,1.387) | 0.857(0.577,1.274) | 0.654(0.486,0.880) |
| Smoking status |  |  |  |  |  |
| Smoking | 0.802(0.687,0.936) | Ref | 0.862(0.590,1.260) | 0.645(0.420,0.873) | 0.568(0.387,0.833) |
| No-smoking | 0.864(0.783,0.953) | Ref | 0.897(0.655,1.228) | 0.659(0.488,0.888) | 0.731(0.562,0.951) |
| Body Mass Index |  |  |  |  |  |
| <median | 0.813(0.733,0.901) | Ref | 0.809(0.598,1.096) | 0.709(0.529,0.957) | 0.577(0.440,0.756) |
| ≥median | 0.903(0.786,1.037) | Ref | 1.129(0.761,1.666) | 0.759(0.516,0.923) | 0.861(0.699,0.973) |
| Poverty Income Ratio |  |  |  |  |  |
| <median | 0.807(0.727,0.895) | Ref | 1.001(0.754,1.328) | 0.848(0.646,1.115) | 0.636(0.487,0.830) |
| ≥median | 0.872(0.791,0.935) | Ref | 0.732(0.466,1.150) | 0.700(0.480,0.921) | 0.533(0.336,0.847) |

Models were adjusted for age, sex, race, education level, poverty-to-income ratio, BMI, smoking status, alcohol consumption, hypertension, and diabetes.The first column presents hazard ratios per 1-point increase in the Mediterranean Diet Score as a continuous variable. Q1-Q4 represent increasing quartiles of MDS, from lowest to highest.
